# Phenome-wide analysis reveals epistatic associations between *APOL1* variants and chronic kidney disease and multiple other disorders

**DOI:** 10.1016/j.ebiom.2024.105000

**Published:** 2024-02-14

**Authors:** Walt E. Adamson, Harry Noyes, Paul Johnson, Anneli Cooper, Darren G. Monckton, John Ogunsola, Georgia Beckett-Hill, Michael Sullivan, Patrick Mark, Rulan S. Parekh, Annette MacLeod

**Affiliations:** a School of Biodiversity, One Health, and Veterinary Medicine, University of Glasgow, United Kingdom; b Wellcome Centre for Integrative Parasitology, University of Glasgow, United Kingdom; c TrypanoGEN+ Research Group, Uganda, Member of the H3Africa Consortium, South Africa; d Centre for Genomic Research, University of Liverpool, United Kingdom; e School of Molecular Biosciences, University of Glasgow, United Kingdom; f School of Cardiovascular and Metabolic Health, University of Glasgow, United Kingdom; g Women’s College Hospital, Hospital for Sick Children and University of Toronto, Canada

**Keywords:** APOL1, Phenome, Chronic kidney disease, UK Biobank, COVID-19

## Abstract

**Background:**

*APOL1* variants G1 and G2 are common in populations with recent African ancestry. They are associated with protection from African sleeping sickness, however homozygosity or compound heterozygosity for these variants is associated with chronic kidney disease (CKD) and related conditions. What is not clear is the extent of associations with non-kidney-related disorders, and whether there are clusters of diseases associated with individual *APOL1* genotypes.

**Methods:**

Using a cohort of 7462 UK Biobank participants with recent African ancestry, we conducted a phenome-wide association study investigating associations between individual *APOL1* genotypes and conditions identified by the International Classification of Disease phenotypes.

**Findings:**

We identified 27 potential associations between individual *APOL1* genotypes and a diverse range of conditions. G1/G2 compound heterozygotes were specifically associated with 26 of these conditions (all deleteriously), with an over-representation of infectious diseases (including hospitalisation and death resulting from COVID-19). The analysis also exposed complexities in the relationship between *APOL1* and CKD that are not evident when risk variants are grouped together: G1 homozygosity, G2 homozygosity, and G1/G2 compound heterozygosity were each shown to be associated with distinct CKD phenotypes. The multi-locus nature of the G1/G2 genotype means that its associations would go undetected in a standard genome-wide association study.

**Interpretation:**

Our findings have implications for understanding health risks and better-targeted detection, intervention, and therapeutic strategies, particularly in populations where *APOL1* G1 and G2 are common such as in sub-Saharan Africa and its diaspora.

**Funding:**

This study was funded by the 10.13039/100010269Wellcome Trust (209511/Z/17/Z) and H3Africa (H3A/18/004).

**Research in context**

**Evidence before this study**

People of recent African origin are disproportionately affected by chronic kidney disease and other kidney-related conditions. This excess risk has been, in part, attributed to two independent variants in the apolipoprotein L1 (*APOL1*) gene known as G1 and G2, which are common in sub-Saharan Africa and its diaspora, but rare or absent in other populations. Heterozygosity for G1 or G2 has been shown to be associated with protection from African sleeping sickness: G1 is associated with decreased disease severity in *Trypanosoma brucei gambiense* infection, while G2 prevents infection by *T. brucei rhodesiense*. Despite the association of different genotypes with distinct phenotypes regarding sleeping sickness, association studies examining G1 and G2 in kidney disease and related conditions have often grouped the two variants together as recessively ‘high-risk’.

**Added value of this study**

In light of the *APOL1* genotype-specific phenotypes observed for African trypanosomiasis we hypothesised that different combinations of *APOL1* variants may be associated with other conditions beyond human African trypanosomiasis and kidney disease. Using data from the UK Biobank, we conducted a phenome wide association study with each *APOL1* genotype in turn and have demonstrated that no association was detected when grouping G1/G1, G1/G2 and G2/G2 risk genotypes together, however, when each genotype was examined individually, a spectrum of 27 potential *APOL1*-associated conditions was detected in addition to kidney disease. We highlight that compound heterozygosity, G1/G2, is associated with the majority of conditions (26/27), all of which were deleterious and that its effect is often masked by the lack of association with G1/G1 and G2/G2. The multi-locus nature of G1/G2 means that these associations may have previously gone undetected in a standard genome-wide association study. In addition, detailed examination of association with chronic kidney disease indicators revealed that different genotypes are associated with different measures of kidney function: G1 homozygosity with proteinurea, G2 with glomerular filtration rate, and G1/G2 with proteinurea and end stage kidney disease.

**Implications of all the available evidence**

The findings presented here highlight the need to consider *APOL1* genotypes individually rather than classifying G1/G2 alongside G1 homozygotes and G2 homozygotes collectively as ‘two-risk-variant genotypes’. This has implications for future association studies, as well as for investigating the molecular mechanisms causing cell injury in *APOL1*-associated diseases. The G1/G2 genotype is carried by millions of people worldwide, and our observations have the potential to significantly impact the way that health risks are understood, particularly in populations where *APOL1* G1 and G2 are common such as in sub-Saharan Africa and its diaspora.

## Introduction

People of recent African origin are disproportionately affected by chronic kidney disease (CKD).^1^ This excess risk has been substantially attributed to the carriage of two independent variants in the apolipoprotein L1 (*APOL1*) gene referred to as G1 and G2.^2^ G1 and G2 are common in sub-Saharan Africa and its diaspora, with estimated allele frequencies of 21% and 13%, respectively, in African Americans,^2^ and up to 49% and 21% in West Africa.^3^ G1 and G2 are absent or occur at very low frequency in non-African-derived populations, consistent with the hypothesis that these variants arose in West Africa only 10,000 years ago and were subject to selection in that population^2^ prior to spreading to much of sub-Saharan African and its recent diaspora.

G1 (amino acid substitutions S342G and I384M) and G2 (deletion of N388 and Y389) are both found in the same domain at the C-terminus of *APOL1* only 20 bp apart, but are present on separate haplotypes^2^ and are in complete linkage disequilibrium with each other such that haplotypes with both G1 and G2 alleles are either very rare or absent. Haplotypes containing neither G1 nor G2 are termed G0. G1 and G2 are collectively considered to be high-risk variants for deleterious kidney phenotypes: carriage of two alleles is associated with a spectrum of CKD conditions, including focal segmental glomerulosclerosis, hypertension-associated kidney failure, and HIV-associated nephropathy.^2^^,^^4^, ^5^, ^6^ These associations are strongest in severe forms of nephropathy, and weaker with mild disease, indicating that APOL1 might contribute to more rapid disease progression.^7^ Recently, in SARS-CoV-2-infected African Americans, carriage of two high-risk *APOL1* variants has been associated with collapsing glomerulopathy,^8^ acute kidney injury (AKI), persistent AKI, and requirement for kidney replacement therapy.^9^ Among patients with COVID-19 disease, carriage of two high-risk variants was associated with increased AKI severity and death.^10^ Associations between high-risk *APOL1* variants and non-kidney-specific phenotypes have also been described, including a range of cardiovascular outcomes, however, inconsistently.^11^ Studies examining associations with high-risk *APOL1* variants focused primarily on African American cohorts: comparable data for other populations with recent African ancestry is limited.

Previously, a phenome-wide study identified conditions associated with the carriage of two-variant *APOL1* genotypes^12^ associated with conditions recorded via International Classification of Diseases, Ninth Revision (ICD-9) and Tenth Revision (ICD-10) codes. Using stringent criteria, they did not detect associations with non-kidney traits, and concluded that *APOL1* likely only operates in kidney-specific pathways.

*APOL1* is found only in humans and few higher primates and is expressed as both a secreted high-density-lipoprotein-associating form by the liver, and as intracellular forms by a variety of cell types, including endothelial cells. Prior to its associations with non-communicable diseases, secreted APOL1 had been identified as the trypanolytic protein: a pore-forming serum protein that lyses protozoan trypanosome parasites, protecting humans from infection by many trypanosome species.^3^ The two subspecies of *T. brucei* that infect humans, *T.b. rhodesiense* and *T.b. gambiense*, which cause human African trypanosomiasis, have developed specific mechanisms for avoiding lysis by APOL1, either by binding, avoiding, or degrading the lytic protein.^13^ Studies examining the effect of the *APOL1* variants in *T.b. gambiense* and *T.b. rhodesiense* infections have highlighted differences between the genotypes. Resistance to *T.b. rhodesiense* infection was associated solely with the G2 variant but not G1, while in *T.b. gambiense* infections, carriage of G1 and G2 were associated with decreased and increased risk of severe disease, respectively.^3^ Due to the mainly protective association between *APOL1* risk alleles and human African trypanosomiasis it has been proposed that trypanosomes are the selective agent for *APOL1* G1 and G2 alleles in African populations. This is analogous to the classic example of *Plasmodium* selection for the sickle-cell allele of beta globin in individuals with sickle-cell trait.^14^

Association studies examining *APOL1* G1 and G2 have often grouped the two variants together as recessively ‘high-risk’, however for some conditions the different genotypes have distinct phenotypes as described above for African sleeping sickness. In a cohort of African American patients on long-term haemodialysis, different *APOL1* genotypes were associated with different rate of progression to haemodialysis.^15^ Recently, the G1/G1 genotype (but not G1/G2 or G2/G2) was associated with proteinuria in a cross-sectional population-based cohort in sub-Saharan Africa.^16^

In light of the *APOL1* genotype-specific phenotypes observed for African trypanosomiasis and the spectrum of kidney-related conditions associated with *APOL1*, we hypothesised that different combinations of *APOL1* variants may be associated with other conditions beyond human African trypanosomiasis and CKD. We assessed the association of *APOL1* variants in a phenome wide study of a large population from the UK Biobank, a large-scale biomedical database and research resource containing genetic, lifestyle, and health information from half a million participants from across the UK.^17^ Using indicators of CKD as a covariate (alongside other appropriate covariates), we performed a phenome-wide screen. We show that G1 and G2 are not equivalent ‘high-risk’ variants and that the G1/G2 compound heterozygous genotype stands out as being particularly associated with deleterious outcomes in a far wider range of conditions than previously reported. In addition, detailed examination of association with CKD indicators revealed that G1/G1, G2/G2 and G1/G2 genotypes all display associations with distinct CKD-related phenotypes.

## Methods

### Study design and participants

The UK Biobank is a prospective cohort study of 502,460 adults aged 40–69 years at enrolment between 2006 and 2010 from 22 assessment centres across the United Kingdom.^17^ At the baseline study visit, participants underwent nurse-led interviews and completed detailed questionnaires about medical history, medication use, sociodemographic factors, and lifestyle in addition to a range of physical assessments and provided blood and urine at the baseline visit. The UK Biobank study was approved by the North-West Multi-Centre Research Ethics Committee, and all participants provided written informed consent.

### Genotyping

*APOL1* genotypes were obtained from the UK Biobank which used a custom Affymetrix array for the G1 (rs73885319) and K (rs73885316) alleles. G2 (rs71785313) genotypes were imputed by the UK Biobank.^17^ Briefly: phasing of autosomes was carried out with Shapeit3; imputation was carried out using the IMPUTE2 algorithm with a reference panel created from the merger of the UK10K reference panel with the 1000 Genomes Project phase 3 panel. This reference panel includes SNPs, short indels, and larger structural variants, and consists of 87,696,888 bi-allelic variants in 12,570 haplotypes. QCTOOL was used to calculate the minor allele frequency (MAF) and imputation information score of each imputed variant.

**Fig. 1:**
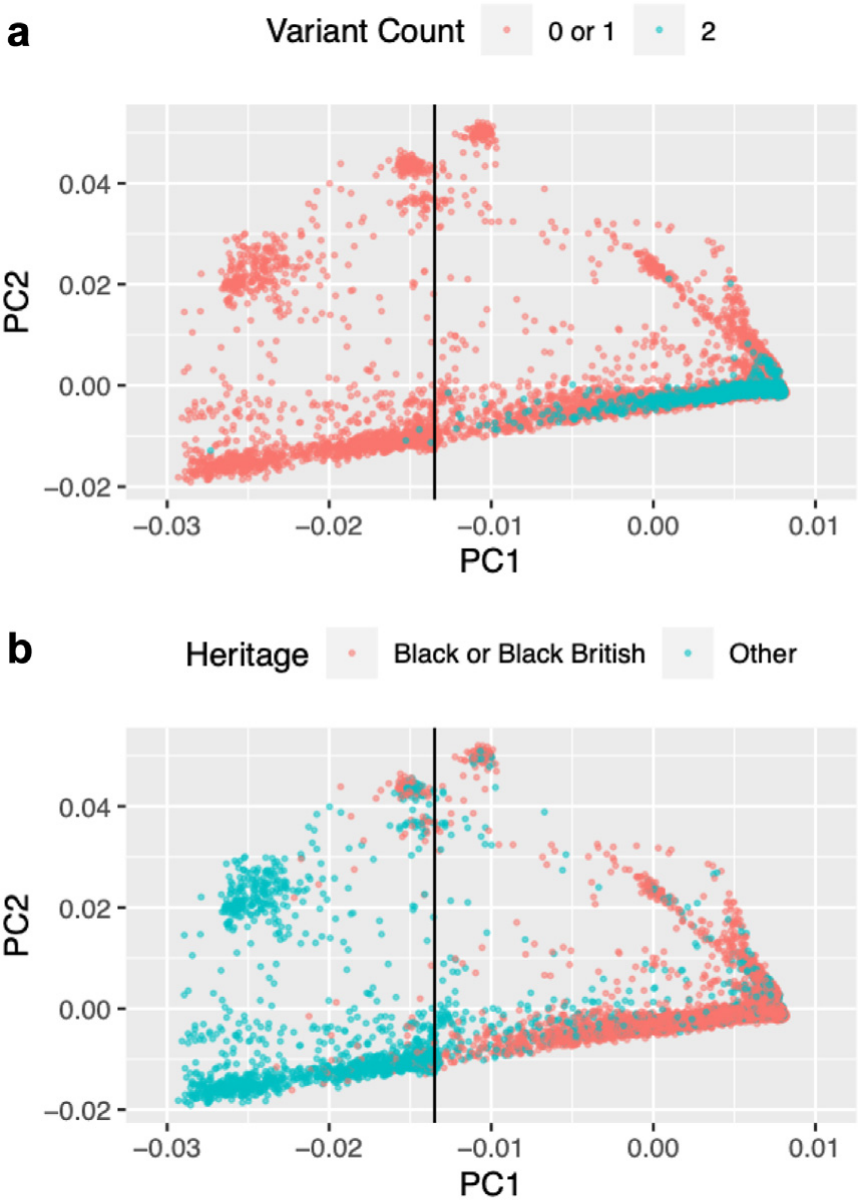
PCA plots of principal components calculated from Affymetrix genotype data from the 10,179 participants that had UK Biobank PC1 > 100 and PC2 > 0. (a) Participants were classified by whether they have a two-risk-variant *APOL1* genotype. (b) Participants were classified by their self-declared ethnicity: Black or Black British participants have UK Biobank ethnicity codes 4001, 4002, or 4003. The vertical line in (a) indicates the cut off used to select participants. Everyone to the right of the line was included.

As *APOL1* G1 and G2 are predominantly found in people with recent African ancestry, we selected UK Biobank participants on the basis of genetic evidence of African ancestry using a two-step process. We inspected a plot of principal components (PC) calculated from genetic data of all UK participants^17^ and identified a cohort 10,179 individuals with PC1 > 100 and PC2 > 0. We then used the UK Biobank whole genome Affymetrix genotype data to calculate principal components for this cohort after filtering to remove linked SNPs. Inspection of a plot of the new PC1 and PC2 (Fig. 1) identified a core set of participants with PC1 > −0.0135, which captured 1117 (93.1%) of the 1200 individuals in the UK Biobank with a two-risk-variant APOL1 genotype, enabling comparison of these individuals with UK Biobank participants who were of the most similar ancestry. Notably, PC1 > −0.0135 also closely demarcates individuals who self-declare Black or Black British ethnicity from those who identify as other ethnicities (selection of PC1 > −0.0135 captures 97.5% of participants with self-declared Black or Black British ethnicity). The data was further filtered to include only participants with complete, unambiguous *APOL1* genotype data, and to remove participants with no UK Biobank blood biochemistry data for cystatin or creatinine (required for the calculation of estimated glomerular filtration rate). The final cohort for analyses described here included 7462 UK Biobank participants.

### Identification of hospital inpatient diagnoses

Records for hospital inpatients and deaths were identified via UK Biobank tables ‘hesin_diag’ and ‘death_cause’. The International Classification of Diseases is a system of diagnostic codes for classifying factors relating to healthcare such as diseases, symptoms, abnormal findings, social circumstances, and external causes of injury. International Classification of Diseases, Ninth and Tenth Revisions (ICD-9 and ICD-10)^18^ codes were used to identify hospital inpatient diagnoses. The UK Biobank contains ICD-9 and ICD-10 codes at four levels of increasing disease specificity (Chapters (Supplementary Table S1), Level 1, Level 2, and Level 3). Level 2 ICD-9 and ICD-10 codes (identified by a letter followed by two digits) were selected as optimal for analyses described here: enabling identification of associations with specific conditions, while ensuring that each code examined had sufficient statistical power.

### Definitions of chronic kidney disease and associated conditions

Indicators of CKD at UK Biobank enrolment were defined in accordance with guidelines from the Kidney Disease: Improving Global Outcomes clinical practice guidelines^19^^,^^20^: either an elevated urinary albumin:creatinine ratio (uACR > 3 mg/mmol), or decreased estimated glomerular filtration rate (eGFR < 60 mL/min/1.73 m^2^). In the UK, clinical diagnosis of CKD requires confirmation of elevated uACR or decreased eGFR in repeat sampling three months apart,^20^ however the UK Biobank provided urine samples at a single time point. eGFR at enrolment was calculated using the CKD-EPI 2021 creatinine and creatinine-cystatin C equations^21^ (which are not adjusted for race), using data collected by the UK Biobank at registration (Supplementary Table S2): age when attended assessment centre, sex, creatinine, and cystatin C. In all analyses, CKD identified by low eGFR means that eGFR was < 60 by either one or both of the two equations. Urinary albumin:creatinine ratio at enrolment was calculated using UK Biobank data fields for microalbumin in urine and creatinine (enzymatic) in urine. End stage kidney disease (ESKD) as of September 2022 was defined as reaching CKD stage G5 or the requirement for kidney replacement therapy, using ICD-10 codes or Office of Population Censuses and Surveys Classification of Surgical Operations and Procedures, Version 4 (OPCS4) codes as described in the UK Biobank’s Definitions of End Stage Renal Disease^22^ (Supplementary data: Identification of end stage kidney disease). Participants were considered hypertensive on UK Biobank registration if they met at least one of the following criteria: self-reported hypertension; prescription of one or more antihypertensive medications for cholesterol, blood pressure, diabetes, or taking exogenous hormones; recording during registration of a systolic blood pressure of > 140 mmHg; recording during registration of diastolic blood pressure of > 90 mmHg (Supplementary Table S2). Participants were considered to have diabetes on UK Biobank registration if they self-reported diabetes or were taking one or more of the following diabetes medications: insulin, gliclazide, glimepiride, tolbutamide, pioglitazone, rosiglitazone, repaglinide, or nateglinide (Supplementary Table S2).

### Covariates

For the phenome-wide screen, age, sex, Townsend deprivation index, principal components 1–4, and CKD (*i.e.,* elevated albumin:creatinine ratio, **or** decreased glomerular filtration rate **or** algorithmically-defined end stage kidney disease) were selected as covariates. CKD was included as a covariate in order to identify associations with *APOL1* genotypes that were not mediated by kidney disease. For examining associations with CKD, covariates were selected based on previously-identified risk factors: age, sex, body mass index, Townsend deprivation index, principal components 1–4, hypertension, and diabetes (Supplementary Table S2).

### Association analysis

The primary exposure variables were the six observed combined G0, G1 and G2 *APOL1* genotypes (Table 1). Only ICD-9 and ICD-10 codes which were assigned to at least 50 participants were retained for analysis. Conditions which affected less than 50 participants were excluded from the analysis since there would be limited power to detect associations with rarer genotypes such as G2/G2. ICD-9 and ICD-10 codes that did not relate to specific diseases and conditions (Chapters XVIII-XXI) were excluded from the analysis. Firth’s bias-reduced logistic regression as implemented in R^23^ was used to control for separation since numbers of some genotypes are expected to be low in some tests, particularly where small numbers of participants had a particular condition. All statistical tests were 2-sided, where a p < 0.05 was considered statistically significant for the primary outcome.

**Table 1:** Haplotype frequencies at the *APOL1* G1 and G2 loci in the UK Biobank cohort (n = 7462).

Genotype name	Haplotypes G1 locus/G2 locus		Variant copy number	G1 copy number	G2 copy number	Number in cohort (% of total) *females/males*	Mean age (median, interquartile range)
G0/G0	AT AT	TTATAA TTATAA	0	0	0	2853 (38.2%) *1638/1215*	51.3 (50, 45–57)
G0/G1	AT GG	TTATAA TTATAA	1	1	0	2273 (30.5%) *1309/964*	52.2 (51, 46–58)
G0/G2	AT AT	TTATAA 6 bp deletion	1	0	1	1219 (16.3%) *701/518*	52.0 (51, 45–58)
G1/G1	GG GG	TTATAA TTATAA	2	2	0	644 (8.6%) *379/265*	52.3 (51, 46–58)
G1/G2	GG AT	TTATAA 6 bp deletion	2	1	1	320 (4.3%) *181/139*	52.0 (51, 45–58)
G2/G2	AT AT	6 bp deletion 6 bp deletion	2	0	2	153 (2.1%) *100/53*	51.6 (50, 45–57)

Genotypes with G1 and G2 on the same haplotype are theoretically possible but have not been observed.

The primary objective of this analysis was to identify which of the six observed *APOL1* compound genotypes had an excess of associations with ICD-9 and ICD-10 codes, rather than demonstrating associations with any specific condition. It is an exploratory study identifying potential associations that could subsequently be investigated in additional datasets, and for this purpose, the benefit of including a proportion of false positives outweighed the cost of omitting false negative. As a result, a relatively relaxed false discovery rate (FDR) of 20% was chosen. The Q value^24^ package in R was used to calculate FDR.

### Ethical approval

Access to the UK Biobank data was granted for this work under UK Biobank application number 66821.

### Role of funder

The funders of the study had no role in the study design, data analysis, data interpretation, or writing of this manuscript.

## Results

### Identification of the cohort

Using principal component data, we identified 7462 UK Biobank participants as having recent African ancestry, unambiguous *APOL1* genotype data, and UK Biobank blood biochemistry data for creatinine and cystatin. This cohort accounted for 93% of participants with G1/G1, G1/G2, or G2/G2 genotypes, 81% of participants who were G0/G1, and 80% of those who were G0/G2. Genotype frequencies for the cohort are shown in Table 1. The allele frequencies in the cohort for G0, G1, and G2 were 62%, 26%, and 12%, respectively.

### Phenome-wide associations with *APOL1* variants

In order to identify which, if any, of the six observed *APOL1* genotypes had associations with diseases, we examined all hospital inpatient diagnoses as defined by ICD-9 and ICD-10 codes. The UK Biobank contains ICD-9 and ICD-10 codes at four levels (Chapter, Level 1, Level 2, and Level 3) of increasing disease specificity. We excluded codes that affected fewer than 50 participants from the analysis since there would be very limited statistical power to detect associations between rarer *APOL1* genotypes and such conditions. We identified 217 Level 2 ICD-9 and ICD-10 codes that were recorded for at least 50 cohort members. Five models of association were examined: (i) whether conditions were associated with any of the five *APOL1* risk variant genotypes; (ii) whether conditions were associated with G1 in either a dominant or a recessive model; (iii) whether conditions were associated with G2 in either a dominant or a recessive model; (iv) whether G1 and G2 were equivalent, and conditions were associated with carriage of either risk allele in either a dominant or a recessive model; (v) whether associations with either risk allele were dose-dependent, and therefore stronger with an increasing number of risk alleles (Table 2).

**Table 2:** Models of association considered in the phenome-wide screen, and number of potential associations identified by each model.

Analysis model	Genotype/grouping	Comparator	Level 2 codes: p < 0.05 and FDR < 20%
Genotype	G0/G1	G0/G0	0
Genotype	G0/G2	G0/G0	**1**
Genotype	G1/G1	G0/G0	0
Genotype	G1/G2	G0/G0	**26**
Genotype	G2/G2	G0/G0	0
G1 dominant	1xG1 (G0/G1, G1/G2)	0xG1 (G0/G0, G0/G2, G2/G2)	0
G1 recessive	2xG1 (G1/G1)	0xG1 (G0/G0, G0/G2, G2/G2)	0
G2 dominant	1xG2 (G0/G2, G1/G2)	0xG2 (G0/G0, G0/G1, G1/G1)	0
G2 recessive	2xG2 (G2/G2)	0xG2 (G0/G0, G0/G1, G1/G1)	0
Risk allele dominant	1 variant (G0/G1, G0/G2)	0 variants (G0/G0)	0
Risk allele recessive	2 variants (G1/G1, G1/G2, G2/G2)	0 variants (G0/G0)	0
G1 additive	G1 count	0 variants (G0/G0)	0
G2 additive	G2 count	0 variants (G0/G0)	0
Risk allele additive	Risk allele count	0 variants (G0/G0)	0

Dominant models were where one risk allele was sufficient to produce phenotype, recessive models where two risk alleles were required to produce phenotype, and additive models where risk of phenotype was proportional to the number of variant alleles present. Numbers in bold indicate analysis models in which an association was detected.

**Fig. 2:**
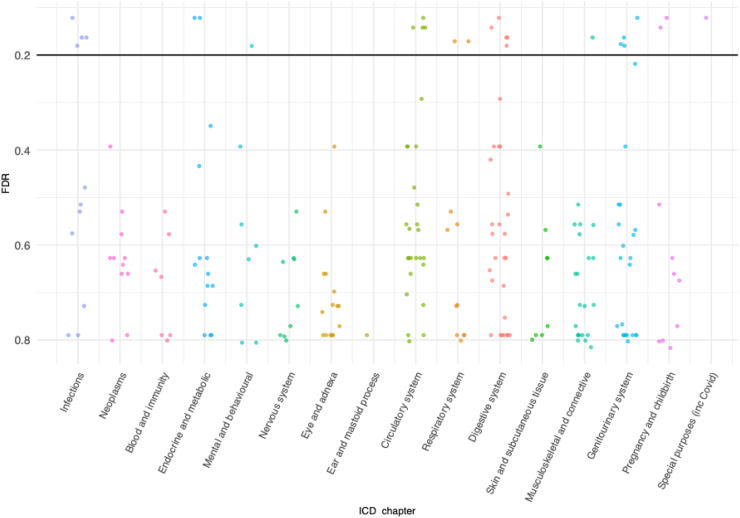
False discovery rate values for association between Level 2 ICD-10 codes and *APOL1* G1/G2 genotype. Horizontal line indicates the threshold that was used for the false discovery rate (20%) for a potentially significant association. Colouring is used to demarcate ICD chapters. Codes which were recorded for at least 50 cohort members were tested. The number of codes tested in each chapter is shown in Supplementary Table S4.

Each phenotype was tested for an association with *APOL1* genotype using logistic regression with age, sex, Townsend deprivation index, evidence of chronic kidney disease (eGFR < 60 mL/min/1.73 m^2^ or uACR > 3 mg/mmol or algorithmically-defined ESKD), and principal components 1–4 used as covariates. A false discovery rate was used to adjust for multiple testing. The aim of the study was to identify the haplotype combinations which had an excess of associations with ICD-9 and ICD-10 codes, rather than demonstrating associations with any specific condition, making it appropriate to use a relatively relaxed FDR threshold of 20%, in a similar approach to other association studies.^25^^,^^26^ Twenty-seven potential associations (p < 0.05 and FDR < 20%) were detected (Table 2, Fig. 2, Supplementary Table S3). One specific risk compound genotype dominated this analysis, with 26 (96.3%) of the associations linked to the G1/G2 genotype: significantly more than any other genotype, (Fisher’s exact test, p = 2 × 10^−7^); particularly remarkable given that 644 and 320 participants had G1/G1 and G1/G2 genotypes respectively, indicating a substantially greater power to detect associations with G1/G1 than G1/G2 (Supplementary data: Sensitivity Analysis). Conversely, there were only 153 participants with the G2/G2 genotype (Table 1), and it is possible that the complete absence of associations with G2/G2 with FDR < 20% is due to lack of statistical power (Supplementary data: Sensitivity Analysis). Country of origin of the participants (UK and Ireland versus elsewhere) had negligible effect on p-values and odds ratios in this analysis (data not shown).

The only non-G1/G2 association (the association between the G0/G2 genotype and code H35 (other retinal disorders)) is shown in Supplementary Fig. S1 and Table S3. Notably, no potential associations were detected for the high-risk variants when considered collectively. Odds ratios indicated that all 27 potential associations were deleterious, with increased rates of hospitalisation among cohort members with the variant genotype. In analyses that considered G1 and G2 in dominant and recessive models, and in analyses that grouped G1 and G2 together as equivalent risk alleles, no associations were detected.

We then used z-score tests to examine whether the 26 associations with G1/G2 genotype were randomly distributed across coding chapters, or whether specific chapters were over- or under-represented. ICD-10 code U07 predominantly represented people who had been hospitalised for a cause related to COVID-19. It was the only chapter 22 (codes for special purposes) code analysed in this study. As a result, U07 was grouped with chapter I (certain infectious and parasitic diseases) codes (A00-B99) in this analysis. This chapter I/U07 grouping was over-represented in G1/G2 associations, with associations detected in 5/11 codes (Z-test, 45.5%, p = 0.002) (Supplementary Table S4). No coding chapters were significantly under-represented.

Finally, we tested whether any of the *APOL1* genotypes were associated with an excess of ICD-9 and ICD-10 codes using logistic regression with age, sex, Townsend deprivation index, evidence of chronic kidney disease, and principal components 1–4 used as covariates. UK Biobank participants in our cohort had a mean of 6.9 Level 2 codes assigned to them. In this analysis the G1/G2 genotype again emerged as distinct with a significantly higher average number of codes (8.7) than that recorded for participants with the G0/G0 genotype (6.5, p = 0.0003). No such differences were observed for any other *APOL1* genotypes containing risk variants (Supplementary Table S5).

### Epistasis partially explains the excess of associations with the G1/G2 genotype

The excess of associations with the G1/G2 genotype and the absence of associations with more frequent *APOL1* genotypes could be explained by an epistatic interaction between the G1 and G2 loci. Our primary analysis model that considered each of the six observed *APOL1* genotypes as single variables did not separate the effects of G1 and G2 from an interaction effect. We explicitly tested for epistasis by examining an equivalent model (Model 2) that considered genotypes at the G1 locus, G2 locus genotypes, and the G1/G2 interaction as separate independent variables. Using Model 2, six ICD-9 and ICD-10 codes had associations with the G1/G2 interaction (Supplementary Table S6). Although this alternative model identified fewer associations, it also has less statistical power to do so as indicated by a larger mean standard error for the G1/G2 interaction in Model 2 (0.43) than the G1/G2 genotype in primary analysis model (0.33) (paired t-test, p = 9 × 10^−9^) (Supplementary Material: Interaction analysis and epistasis). Furthermore, in our primary analysis model, the associations observed for the G1/G2 genotype are not only due to the G1/G2 epistatic interaction, but also a result of the effects of being heterozygous at the G1 locus or being heterozygous at the G2 locus. Each of these effects may be too small to be significant, but are significant when combined. Therefore, Model 2 provides explicit evidence for epistasis between G1 and G2 driving associations in six ICD-9 and ICD-10 codes and evidence for epistasis contributing to other associations.

### Associations between *APOL1* G1/G2 and hospitalisation due to infectious diseases

The phenome-wide screen indicated an association between the G1/G2 genotype and a range of hospital inpatient diagnoses, with an overrepresentation of codes related to infectious diseases (chapters I and XXII), including COVID-19. Due to statistical power considerations, the phenome-wide screen was limited to conditions in which at least 50 cases within the cohort were recorded. Many individual infectious diseases did not reach this threshold. In order to examine associations between *APOL1* genotype and hospitalisation from any infectious disease, we performed an additional logistic regression using age, sex, Townsend deprivation index, chronic kidney disease, and principal components 1–4 as covariates. ICD-9 and ICD-10 codes in Chapter I (A00–B99), plus codes J00–J06 (acute upper respiratory infections), J09-J18 (influenza and pneumonia), and J20–J22 (other acute lower respiratory infections) were considered to be indicative of hospitalisation as a result of an infectious disease. ICD-10 code U07 was excluded from the analysis: the phenome-wide screen had already identified an association between the G1/G2 genotype and hospitalisation as a result of COVID-19, and the volume of data on COVID-19 compared to other infectious diseases had the potential to skew the analysis. The G1/G2 genotype was associated with an increased risk of hospitalisation as a result of any (non-Covid-19) infectious disease (Firth’s logistic regression, OR = 1.4, 95%CI: 1.1–1.9, p = 0.007). No such association was detected for any other *APOL1* genotype containing risk variants (Table 3). No association between *APOL1* genotype and death as a result of non-Covid-19 infectious diseases was detected.

**Table 3:** Association of risk of hospitalisation and death as a result of a (non-COVID-19) infectious disease (defined as ICD-9 and ICD-10 codes A00–B99, J00–J06, J09–J18, and J20–J22) with *APOL1* genotypes compared to G0/G0, adjusted for age, sex, Townsend deprivation index, and genetic principal components 1–4.

Genotype	n (total)	n (hospitalisation) (%)	Odds ratio	p	n (death) (%)	Odds ratio	p
G0/G0	2853	666 (23.3%)	1.0 (ref)		10 (0.4%)	1.0 (ref)	
G0/G1	2273	517 (22.7%)	0.9 (0.8–1.1)	0.35	10 (0.4%)	1.0 (0.4–2.5)	0.95
G0/G2	1219	269 (22.1%)	0.9 (0.8–1.1)	0.27	5 (0.4%)	1.0 (0.3–2.8)	0.94
G1/G1	644	148 (23.0%)	0.9 (0.7–1.1)	0.37	1 (0.2%)	0.5 (0.5–2.2)	0.39
**G1/G2**	320	**101 (31.6%)**	**1.4 (1.1–1.9)**	**0.007**	1 (0.3%)	0.9 (0.1–4.0)	0.91
G2/G2	153	33 (21.6%)	0.9 (0.6–1.3)	0.55	0 (0.0%)	0.8 (0.0–6.4)	0.88

p values < 0.05 are shown in bold. The G1/G2 genotype was associated with hospitalisation as a result of a (non-COVID-19) infectious disease.

ICD code U07 indicates hospitalisation due to a factor related to COVID-19, while Level 3 code U071 indicates specifically that the participant had tested positive for SARS-CoV-2 and had been hospitalised as a result. The phenome-wide screen identified an association between the G1/G2 genotype and code U07 (Firth’s logistic regression, OR = 2.5, 95% CI: 1.4–4.2, p = 0.002) Chi-squared tests confirmed that this association was not a result of increased rates of testing (p = 0.08) or test positivity (p = 0.32) among participants with that genotype. Of the 178 cohort members who had received code U07, the majority (154, 86.5%) had tested positive for SARS = CoV-2. Focusing solely on code U071 strengthened the association: the G1/G2 genotype was associated with testing positive for SARS-CoV-2 and being hospitalised as a result (Firth’s logistic regression, OR = 2.4, 95% CI: 1.2–4.1, p = 0.01) (Table 4). Furthermore, the G1/G2 genotype was associated with death following a positive test for SARS-CoV-2 (Firth’s logistic regression, OR = 6.6, 95% CI: 2.5–16.7, p = 0.0003). No such associations were detected for any other *APOL1* genotypes containing risk variants.

**Table 4:** Association of risk of hospitalisation and death as a result of COVID-19 (defined as ICD-10 code U071) with *APOL1* genotypes compared to G0/G0, adjusted for age, sex, Townsend deprivation index, and genetic principal components 1–4.

Genotype	n (total)	n (hospitalisation) (%)	Odds ratio	p	n (death) (%)	Odds ratio	p
G0/G0	2853	54 (1.9%)	1.0 (ref)		14 (0.5%)	1.0 (ref)	
G0/G1	2273	49 (2.2%)	1.1 (0.7–1.6)	0.72	12 (0.5%)	1.3 (0.6–2.9)	0.54
G0/G2	1219	23 (1.9%)	1.0 (0.6–1.6)	0.87	5 (0.4%)	1.0 (0.3–2.6)	0.99
G1/G1	644	11 (1.7%)	0.8 (0.4–1.6)	0.58	2 (0.3%)	0.9 (0.2–3.3)	0.93
**G1/G2**	320	**15 (4.7%)**	**2.3 (1.2–4.1)**	**0.01**	**8 (2.5%)**	**6.6 (2.5–16.7)**	**0.0003**
G2/G2	153	2 (1.3%)	0.8 (0.2–2.4)	0.74	1 (0.7%)	2.4 (0.3–10.1)	0.38

p values < 0.05 are shown in bold. The G1/G2 genotype was associated with hospitalisation and death as a result of a COVID-19.

### Associations between *APOL1* variants and chronic kidney disease

Data from the phenome-wide screen demonstrated that different *APOL1* genotypes had distinct patterns of association. We therefore tested whether these differences also applied to CKD: the disease in which associations between *APOL1* genotypes and pathology were first discovered. Within the cohort, 808 individuals (10.8%) were identified as having at least one indicator of CKD on UK Biobank registration (*i.e.,* eGFR (creatinine) < 60 mL/min/1.73 m^2^ **or** eGFR (creatinine-cystatin C) < 60 mL/min/1.73 m^2^ **or** uACR > 3 mg/mmol) (Supplementary Table S7). In the following analyses, individuals with eGFR < 60 mL/min/1.73 m^2^ by either the creatinine or the creatinine-cystatin C equation were considered to have CKD. Both eGFR and uACR indicators of CKD were present in 72 individuals (1.0% overall, 8.8% of those with CKD). We investigated the association between carriage of two high-risk variants (genotypes G1/G1, G1/G2, and G2/G2) in our UK Biobank cohort, in a similar manner to previous studies on African Americans,^2^ using the first four principal components as covariates along with previously-associated CKD risk factors^27^ (age, sex, body mass index, Townsend deprivation index, hypertension, and diabetes). Associations were detected between carriage of two high-risk variants and CKD (as defined as either uACR > 3 mg/mmol or eGFR < 60 mL/min/1.73 m^2^) as well as uACR > 3 mg/mmol individually. No such association was detected for eGFR < 60 mL/min/1.73 m^2^ (Table 5). Recent evidence suggests that an additional *APOL1* variant, N264K (rs73885316) reduces the cytotoxicity of *APOL1* high-risk variants in HEK293 kidney cells.^28^ This variant was present in 223 cohort members (3.0%) however when it was applied as an additional covariate, it did not affect associations reported here. The N264K variant alone was not associated with any CKD phenotypes examined in this study (data not shown).

**Table 5:** Association of risk indicators of chronic kidney disease with number of *APOL1* risk variants, compared to 0 variants, adjusted for age, sex, body mass index, diabetes, hypertension, Townsend deprivation index, and genetic principal components 1–4.

Genotype	n (total)	uACR > 3 mg/mmol or eGFR < 60 mL/min/1.73m^2^		uACR > 3 mg/mmol		eGFR < 60 mL/min/1.73m^2^	
		Odds ratio (95% CI)	p	Odds ratio (95% CI)	p	Odds ratio (95% CI)	p
0 variants	2853	1.0 (ref)		1.0 (ref)		1.0 (ref)	
1 variant	3492	1.1 (0.9–1.3)	0.52	1.1 (0.9–1.4)	0.18	0.9 (0.7–1.3)	0.69
2 variants	1117	**1.4 (1.1–1.8)**	**0.002**	**1.5 (1.2**–**2.0)**	**0.001**	1.4 (1.0–2.0)	0.05

Genotypes with p values < 0.05 are shown in bold. Carriage of two APOL1 risk variants was associated with having chronic kidney disease risk indicators, consistent with previous studies.^2^ The numbers and percentages are shown in Supplementary Table S7.

We then examined each genotype combination individually, using the same statistical method. First, we demonstrated that the inclusion of *APOL1* G1 and G2 combined genotypes in a logistic regression model significantly improved the fit of the model to the data (ANOVA p = 0.044) and that therefore *APOL1* genotypes contribute to risk of CKD. We tested whether the individual combined genotypes made equivalent contributions to risk of CKD or whether it was important to examine them separately by constraining all alternate genotypes to have the same effect and comparing this model with the full model with one reference genotype and five alternate genotypes. The full model was a significantly better fit to the data (ANOVA p = 0.044), demonstrating that examining individual genotypes is more informative than grouping all two-variant genotypes together. Logistic regression using individual genotype combinations showed that G1/G1 and G1/G2 were associated with having an indicator of CKD at UK Biobank registration (either uACR > 3 mg/mmol or eGFR < 60 mL/min/1.73 m^2^), however no such association was detected for G2/G2 (Table 6). Considering indicators of CKD individually, having uACR > 3 mg/mmol was associated with G1/G1 and G1/G2, while eGFR < 60 mL/min/1.73 m^2^ was associated specifically with G2/G2. By examining each two-risk-variant genotype individually rather than grouping them together, we detect complexities in the relationship between *APOL1* risk variants and CKD, suggesting that APOL1-mediated cell injury in CKD might be a result of more than one genotype-specific molecular pathway.

**Table 6:** Association of risk indicators of chronic kidney disease with *APOL1* genotypes compared to G0/G0, adjusted for age, sex, body mass index, diabetes, hypertension, Townsend deprivation index, and genetic principal components 1–4.

Genotype	n (total)	uACR > 3 mg/mmol or eGFR < 60 mL/min/1.73m^2^		uACR > 3 mg/mmol		eGFR < 60 mL/min/1.73m^2^	
		Odds ratio (95% CI)	p	Odds ratio (95% CI)	p	Odds ratio (95% CI)	p
G0/G0	2853	1.0 (ref)		1.0 (ref)		1.0 (ref)	
G0/G1	2273	1.0 (0.8–1.3)	0.75	1.1 (0.9–1.4)	0.24	0.9 (0.6–1.2)	0.52
G0/G2	1219	1.1 (0.9–1.4)	0.37	1.1 (0.9–1.5)	0.28	1.0 (0.7–1.5)	0.86
**G1/G1**	644	**1.4 (1.1–1.9)**	**0.01**	**1.6 (1.2–2.1)**	**0.003**	1.2 (0.8–1.9)	0.37
**G1/G2**	320	**1.6 (1.1–2.2)**	**0.01**	**1.7 (1.1–2.5)**	**0.01**	1.5 (0.9–2.6)	0.15
**G2/G2**	153	1.2 (0.7–2.0)	0.52	1.0 (0.5–1.8)	0.96	**2.3 (1.1–4.4)**	**0.02**

Genotypes with p values < 0.05 are shown in bold. The numbers of affected participants with each genotype and percentages are shown in Supplementary Table S8.

### Associations between *APOL1* variants and end stage kidney disease

Having identified genotypic associations with indicators of CKD measured at the time of participants’ registration to the UK Biobank, we examined incidences of end stage kidney disease (ESKD) in our cohort recorded by September 2022. ESKD was defined algorithmically according to the UK Biobank’s guidelines,^22^ and based on ICD-10 and OPCS4 codes recorded for each cohort member. There was strong correlation with subsequent ESKD with having eGFR < 60 mL/min/1.73 m^2^ (Firth’s logistic regression, OR = 19.3, 95% CI: 11.0–34.0, p < 1 × 10^−14^) or uACR > 3 mg/mmol (Firth’s logistic regression, OR = 8.3, 95% CI: 5.0–14.2, p < 10^−14^) at UK Biobank recruitment. We then examined genotypic associations with ESKD: an association was detected solely for the G1/G2 genotype (Firth’s logistic regression, OR = 3.2, 95% CI: 1.4–6.8, p = 0.007) (Table 7).

**Table 7:** Association of risk of end stage kidney disease with *APOL1* genotypes compared to G0/G0, adjusted for age, sex, body mass index, diabetes, hypertension, Townsend deprivation index, and genetic principal components 1–4.

Genotype	n (total)	n (end stage kidney disease) (%)	Odds ratio	p
G0/G0	2853	23 (0.8%)		
G0/G1	2273	18 (0.8%)	0.9 (0.5–1.8)	0.84
G0/G2	1219	10 (0.8%)	1.1 (0.5–2.2)	0.91
G1/G1	644	9 (1.4%)	1.5 (0.7–3.3)	0.33
**G1/G2**	320	**10 (3.1%)**	**3.3 (1.5–7.2)**	**0.005**
G2/G2	153	1 (0.7%)	1.3 (0.1–5.2)	0.78

p values < 0.05 are shown in bold.

In analysing the relationship between *APOL1* genotypes and CKD, we identified associations that were not apparent from treating G1 and G2 as equivalent ‘high-risk’ alleles, and that the two-variant *APOL1* genotypes displayed distinct phenotypes in terms of CKD indicators and disease progression.

## Discussion

Relationships between G1 and G2 *APOL1* variants and several non-communicable diseases including chronic kidney disease are well-established in African American populations.^1^ Here, we confirm this association in a population-based cohort of people with recent African ancestry living in the United Kingdom. We show that one specific multi-locus genotype, G1/G2, is associated with multiple disease phenotypes with primarily deleterious outcomes, and that the scope of conditions affected by this particular genotype might be far wider than previously thought. While studies of the relationship between high-risk *APOL1* variants and CKD have largely focused on the number of variants in an individual, with carriage of two variants considered deleterious, we provide evidence that the different combinations of *APOL1* variants are associated with different phenotypes.

Our phenome-wide association study revealed 27 potential associations, all of which were deleterious: far more than would be expected if the potential associations occurred by chance with equal likelihood of positive and negative effects (Binomial p = 7 × 10^−9^). No association was detected when grouping G1/G1, G1/G2 and G2/G2 risk genotypes together. However, when each genotype was examined individually, a spectrum of potential *APOL1*-associated conditions was detected. This revealed that G1/G2 is potentially associated with several disease phenotypes, and that its effect is often masked by the lack of association with G1/G1 and G2/G2. The lack of association when grouping risk genotypes together is consistent with a similar phenome-wide association study of high-risk *APOL1* variants that was conducted using data from 6579 African American participants from Penn Medicine Biobank and Vanderbilt BioVU.^12^ When CKD/kidney failure status (defined as eGFR < 60 mL/min/1.73 m^2^ or at least two ICD-10 codes for dialysis or kidney transplant) was included as a covariate and a stringent Bonferroni correction applied, no non-renal diseases were associated when *APOL1* two-risk-variant genotypes were grouped together. A repeat of this analysis in which APOL1 genotypes are examined individually and a more relaxed multiple testing methodology was deployed would be informative.

Our analysis identified multiple associations with the G1/G2 genotype, despite the greater power to detect associations with the G1/G1 combination than the G1/G2 combination. Twenty-six associations with G1/G2 had FDR < 20% were detected, whereas no associations with G1/G1 had FDR < 20% (Table 2): a statistically significant difference between the two genotypes (Fisher’s exact test, p = 1 × 10^−8^). Although there was less power to detect associations with the G2/G2 haplotype combination the zero associations with G2/G2 with FDR < 20% (Table 2) suggests that this combination may have less impact on disease that G1/G2. Further targeted studies will be required to identify which of these associations are true positives.

Infectious diseases are overrepresented in terms of conditions that we detected as being potentially associated with G1/G2. The risk of hospitalisation or death from COVID-19 is disproportionately higher in people of African ancestry. There is growing evidence that carriage of two high-risk variant *APOL1* alleles is associated with adverse outcomes in COVID-19. In African Americans infected with SARS-CoV-2, carriage of two *APOL1* variants is associated with collapsing glomerulopathy,^8^ acute kidney injury, persistent AKI, and requirement for kidney replacement therapy.^9^ Among African Americans hospitalised with COVID-19, carriage of two high-risk *APOL1* variants has been associated with increased AKI severity and death.^10^ When considering individual genotypes in our study we revealed that associations with these phenotypes were driven primarily by the G1/G2 genotype, and the inclusion of CKD as a covariate suggests a potential non-renal pathway through which G1/G2 affects SARS-CoV-2 infection outcome.

Infectious disease is a major evolutionary driving force of natural selection. The relationship between high-risk *APOL1* variants and human African trypanosomiasis is well-characterised: G2 is associated with protection from *T.b. rhodesiense* infection, but conversely is associated with increased disease severity of *T.b. gambiense*, whereas G1 is associated with milder disease severity in *T.b. gambiense*.^3^ However, the modern-day distribution of G1 and G2 with both variants being at higher frequency in West Africa does not correspond to the current geographic ranges of the two parasite species: *T.b. rhodesiense* being found only in East Africa and *T.b. gambiense* in West and Central Africa. This suggests that factors other than *T. brucei* sub-species might have provided the selection pressure for the allele distribution. Infectious diseases such as malaria, cholera, dengue, and typhoid have affected millions of people in sub-Saharan Africa for centuries and may have driven the selection of *APOL1* variants but are either rare or absent from the cohort described here. As a result, it has not been possible to assess the impact that *APOL1* genotype has on such conditions or identify other conditions which might be involved in positive selection for *APOL1* variants, however the this warrants further investigation, particularly in light of the observed association between the G1/G2 genotype and hospitalisation from all infectious diseases.

Our study extends the range of infectious in which outcomes are associated with *APOL1* genotype to viral pneumonia and gastroenteritis. Previously, carriage of two high-risk *APOL1* variants has been strongly associated with HIV-associated nephropathy (HIVAN) with odds ratios ranging from 29 to 89 (in African American and black South African cohorts of 1378 and 228 participants respectively).^4^^,^^5^ In the South African study, it is notable that although the analysis groups the three two-variant *APOL1* genotypes together, 19/38 (50.0%) of the HIVAN patients are specifically G1/G2.^5^ Although the prevalence of the G1/G2 genotype in the general population from which the cohort was drawn is not clear, a prevalence of 3–5% in other South African cohorts^16^ suggests a potentially substantial overrepresentation of G1/G2 among South African HIVAN patients.

Similar odds ratios for HIVAN have been reported for G1/G1, G1/G2, and G2/G2 in African Americans, and the same study also reported an association between HIVAN and G0/G1.^4^ Carriage of two high-risk APOL1 variants has also been associated with protection from HIV-associated opportunistic infections in a cohort of 2.066 African Americans,^29^ however, in our study we were unable to examine this association in our dataset as the UK biobank did not contain enough cases of opportunistic infections in HIV-infected individuals to make an assessment. Carriage of two high-risk variant alleles has also previously been associated with sepsis in a cohort of 57,000 African Americans (OR = 1.5),^30^ however in our study no association with sepsis was detected.

The phenome-wide screen also identified potential associations between G1/G2 and conditions related to the transport of ions and other metabolites across membranes (Chapter IV, E00–E90). These associations continue to be detected when CKD is included as a covariate. APOL1 forms an ion channel: in human African trypanosomiasis, APOL1 forms pores in parasite membranes, disrupting ionic balance, and causing lysis.^31^ Therefore, the associations with the transport of ions and metabolites are intriguing and is suggestive of a wider role for APOL1 channels.

With regards to CKD, we demonstrated that different genotypes were associated with different disease indicators. G1/G1 was associated with elevated uACR (>3 mg/mmol), G2/G2 was associated with decreased eGFR (<60 mL/min/1.73 m^2^), and G1/G2 is associated with elevated uACR and more rapid progression to ESKD. Elevated uACR and decreased eGFR are markers for CKD progression, and the associations detected with G1/G1 and G2/G2 respectively suggest that evidence of CKD progression might be detected in G1/G1 and G2/G2 participants in future analyses of this dataset. Calculations of eGFR were performed using the CKD-EPI 2021 equations,^21^ which are not adjusted for ancestry: improving accuracy and precision in estimating GFR for black adults.^32^ Notably, in our dataset, no association with decreased eGFR was detected when *APOL1* two-risk-variant genotypes were grouped together: it only became apparent once genotypes where examined individually.

Recently, a similar association between G1/G1 and elevated uACR was also shown in a sub-Saharan African cohort of 10,769 participants.^16^ The authors did not detect any such association with G1/G2, or an association with eGFR for any *APOL1* genotype, however the odds ratio that they reported for the association between G1/G1 and elevated uACR (3.87) was higher than in this study (1.6). Several factors might account for this difference between studies, such as the use of GFR estimating equations having limited validation in sub-Saharan Africa,^16^ or elevated uACR and low GFR being influenced by other genetic or environmental factors that differentiate the UK population of African ancestry from sub-Saharan Africans. Associations between *APOL1* variants and CKD have also been examined in a Nigerian cohort of 1195 participants.^33^ Among HIV-negative participants, the study reported an association between the G1/G1 genotype and CKD (OR = 2.2), however no such association was detected for G2/G2. However, the authors considered the G1 and G2 variants independently, and associations with the G1/G2 genotype were not reported.

**Fig. 3:**
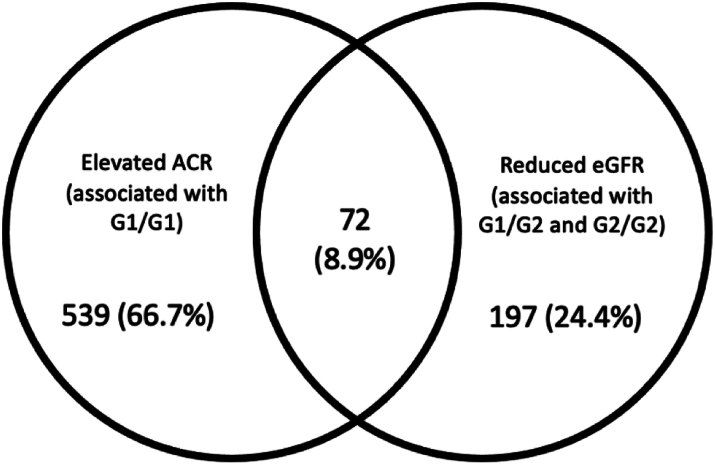
Number of cohort members for whom each indicator of CKD was present at UK Biobank registration. In total, an indicator of CKD was detected in 808 cohort members.

Notably, the majority (91.1%) of the cohort considered to have CKD in this study had just one indicator: *either* reduced eGFR *or* elevated uACR (Fig. 3). The distinct associations observed for each two-high-risk-variant genotype might indicate that cell injury in CKD is modulated by multiple (potentially opposing) genotype-specific molecular pathways that each generate distinct metabolic signatures. An observation that might account for the multiple mechanisms that have been proposed for driving APOL1-related kidney damage.^34^

The membrane-damaging properties of APOL1 might be the common mechanism that explains both the destruction of parasites and kidney injury,^34^ possibly affecting the multiple pathways that have previously been proposed including autophagy, lysosomal permeability, pyroptosis, mitochondrial dysfunction, impairment of vacuolar acidification, activation of stress-related kinases, endoplasmic reticulum stress, and mitophagy.^30^^,^^35^ The number of subunits that comprise an APOL1 pore is unclear, however it has been proposed that the protein acts as a dimer.^36^ One possible hypothesis to explain the wide diversity of diseases associated with *APOL1* G1/G2 is that pores made of heterogenous subunits are either not able to form pores, or form dysfunctional pores, disrupting cellular function and causing adverse phenotypes.

The increased disease burden in G1/G2 compound heterozygotes appears to represent an epistatic interaction, which might explain why it has not been detected in previous genome-wide association studies (GWAS) that focus on associations with individual variants. Since the earliest GWAS, it has been clear that the loci that have been identified only account for a modest proportion of heritability. It has been proposed that this ‘missing heritability’ is due to difficulties in designing well-powered experiments: (i) sample size must be very large to detect loci with small effects; (ii) combinations of variants might have joint effects larger than the sum of their individual effects – epistasis, which would require an exponential increase in sample size to detect in a genome-wide search; (iii) structural variants such copy number variation are not reliably detected by current genotyping methods; (iv) problems with phenotyping.^37^ We found specific evidence for epistasis between G1 and G2 in associations with six ICD-9 and ICD-10 codes, however the specific analysis for interaction used a model that had lower power than the main study. Therefore, it is likely that epistasis contributes to more than six such associations. Therefore, this study provides an example of the potential for epistasis to explain part of the missing heritability. The confirmation of an allelic interaction that is associated with a spectrum of human conditions has potentially far-reaching consequences: there are potentially many other such complex genotypes that impact on human health, and computational methods to simultaneously screen for associations with multiple combinations of alleles would be required.

Associations previously reported between *APOL1* risk alleles and conditions such as CKD have led to calls for the introduction of testing to identify *APOL1* genotype and to minimise the risk of kidney transplant failure. While the potential associations described here require confirmation in other cohorts, they indicate a wide spectrum of conditions that are associated with *APOL1* risk alleles, and particularly with the G1/G2 genotype. Previous studies have highlighted ethical considerations of such testing,^38^ however strong support for testing has recently been reported among African Americans attending hypertension and nephrology clinics in the United States,^39^ and disclosure of high-risk *APOL1* genotypes to hypertension patients has led to reductions in blood pressure and lifestyle changes such as improved dietary and exercise habits.^40^ An affordable, rapid, point-of-care test for *APOL1* genotype would enable testing to be performed in both affluent and resource-poor settings and could have important implications at individual and population level by identifying those who would benefit from targeted early intervention and treatment.

### Limitations of the study

This is an exploratory study that has yielded a set of potential associations that require confirmation in additional datasets. Using a False Discovery Rate of <20% represents a relatively relaxed correction method for multiple testing. Although it is an approach that will yield a proportion of false–positive associations, the frequency of associations detected for G1/G2 compared to any other genotype or analysis model, and the deleterious nature of G1/G2 in all 26 potential associations highlights the uniquely adverse impact that the genotype has on human health. It is possible that the lack of associations with the G2/G2 genotype was due to statistical power limitations. A similar study performed in a cohort containing more participants with the G2/G2 genotype would be informative. The indicators of CKD considered in this study (elevated uACR and decreased eGFR) are based on a single time point at UK Biobank enrolment. Data from additional time points would have strengthened the analyses by: identifying people in whom elevated uACR or decreased eGFR is sustained, and enabling *APOL1* genotypes to be compared in terms of CKD progression rates.

### Conclusions

The compound heterozygous G1/G2 genotype was distinguished as uniquely deleterious in its association with a range of phenotypes. The epistatic nature of a G1/G2 interaction would likely result in such associations going undetected in a standard genome-wide association study. These observations have the potential to significantly impact the way that health risks are understood, particularly in populations where *APOL1* G1 and G2 are common such as in sub-Saharan Africa and its diaspora. The work described here is especially relevant to geographical regions where *APOL1* risk alleles are common such as West and Central Africa and the recent African diaspora which accounts for 140 million individuals worldwide.^41^

## Contributors

WA: conceptualisation, formal analysis; verification of underlying data, writing: original draft. HN: formal analysis, methodology, supervision, verification of underlying data, writing: review and editing. PJ: methodology, supervision, writing: review and editing. AC: writing: review and editing. DM: methodology, writing: review and editing. JO: writing: review and editing. GBH: methodology, writing: original draft. MS: methodology, supervision. PM: methodology, supervision, writing: review and editing. RP: methodology, writing: review and editing. AM: methodology, resources, supervision, writing: original draft; writing: review and editing. All authors have read and approved the final version of the manuscript.

## Data sharing statement

This research has been conducted using data from the UK Biobank, a major biomedical database: www.ukbiobank.ac.uk.

## Declaration of interests

Sullivan has been awarded a Medical Research Council Clinical Research Training Fellowship. Mark has received research funding from Astra Zeneca and Boehringer Ingelheim, consulting fees from GSK, Astellas, Bayer, Astra Zeneca, and Boehringer Ingelheim, payments in honoraria from Astra Zeneca, Boehringer Ingelheim, and Pharmacosmos, and has participated in an advisory board for Novartis. Parekh has received research funding a consulting fees from Vertex Pharmaceuticals. MacLeod has received research funding from Astra Zeneca.
