# Supplementary material for: Phenome-wide analysis reveals epistatic associations between APOL1 variants and chronic kidney disease and multiple other disorders
Source: eBioMedicine. 2024 Feb 14;101:105000. doi: 10.1016/j.ebiom.2024.105000 (PMC10944146; doi:10.1016/j.ebiom.2024.105000)
Supplement: Supplementary data, Fig. S1, and Tables S1–S8 [file mmc1.pdf]

Supplementary data

Chapter	ICD codes	Description
I	A00-B99	Certain infectious and parasitic diseases
II	C00-D48	Neoplasms
III	D50-D89	Diseases of the blood and blood-forming organs and certain disorders involving the immune mechanism
IV	E00-E90	Endocrine, nutritional, and metabolic disorders
V	F00-F99	Mental and behavioural disorders
VI	G00-G99	Diseases of the nervous system
VII	H00-H59	Diseases of the eye and adnexa
VIII	H60-H95	Diseases of the ear and mastoid process
IX	I00-I99	Diseases of the circulatory system
X	J00-J99	Diseases of the respiratory system
XI	K00-K93	Diseases of the digestive system
XII	L00-L99	Diseases of the skin and subcutaneous tissue
XIII	M00-M99	Diseases of the musculoskeletal system and connective tissue
XIV	N00-N99	Diseases of the genitourinary system
XV	O00-O99	Pregnancy, childbirth, and the puerperium
XVI	P00-P96	Certain conditions of the perinatal period
XVII	Q00-Q99	Congenital malformations, deformations, and clinical abnormalities
XXII	U00-U85	Codes for special purposes

Supplementary Table 1: International Classification of Disease codes examined in this study, by chapter. Adapted from World Health Organization *ICD-10 Version:2019*(17).

Data collected at UK Biobank enrolment	UK Biobank data field(s)
Age when attended assessment centre	21003
Blood pressure, diastolic	4079
Blood pressure, systolic	4080
Creatinine	30700
Creatinine (enzymatic) in urine	30510
Cystatin C	30720
Medications taken	6153, 6177, 20003
Microalbumin in urine	30500
Self-reported illness	20002
Sex	31
Standing height	50
Townsend Deprivation Index	189
Weight	21002

Supplementary Table 2: UK Biobank data fields used to identify chronic kidney disease indicators, associated conditions, and covariates as described in Methods.

Genotype/ grouping	ICD code	ICD code descriptor	ICD coding chapter	p-value	Odds ratio (95% CI)	False discovery rate
G0/G2	H35	Other retinal disorders	VII	0·0005	2·1 (1·4-3·2)	0·090
G1/G2	A09	Diarrhoea and gastro-enteritis of presumed infectious origin	I	0·02	1.9 (1·1-3·1)	0·163
G1/G2	B18	Chronic viral hepatitis	I	0·005	4·0 (1·6-9.4)	0·122
G1/G2	B96	Other bacterial agents as the cause of diseases classified to other chapters	I	0·02	1·8 (1·1-2·8)	0·163
G1/G2	B97	Viral agents as the cause of diseases classified to other chapters	I	0·03	2·2 (1·1-4·1)	0·180
G1/G2	E16	Other disorders of pancreatic internal secretion	IV	0·005	2·8 (1·4-5·2)	0·122
G1/G2	E55	Vitamin D deficiency	IV	0·004	2·3 (1·3-4·0)	0·122
G1/G2	F41	Other anxiety disorders	V	0·03	2·0 (1·1-3·6)	0·181
G1/G2	I08	Multiple valve diseases	IX	0·01	2·7 (1·3-5·4)	0·142
G1/G2	I35	Nonrheumatic aortic valve disorders	IX	0·009	3·8 (1·4-9.1)	0·142
G1/G2	I45	Other conduction disorders	IX	0·01	3·3 (1·4-7·3)	0·142
G1/G2	I73	Other peripheral vascular diseases	IX	0·005	2·9 (1·4-5·8)	0·122
G1/G2	J12	Viral pneumonia, not elsewhere classified	X	0·02	2·3 (1·2-4·4)	0·171
G1/G2	J96	Respiratory failure, not elsewhere classified	X	0·02	2·1 (1·1-3·8)	0·171
G1/G2	K22	Other diseases of the oesophagus	XI	0·004	2·6 (1·4-4·7)	0·122
G1/G2	K56	Pancreatic ileus and intestinal obstruction without hernia	XI	0·02	2·3 (1·1-4·4)	0·180
G1/G2	K58	Irritable bowel syndrome	XI	0·02	2·5 (1·2-4·7)	0·163

G1/G2	K59	Other functional intestinal disorders	XI	0·009	1·7 (1·1-2·5)	0·142
G1/G2	K66	Other disorders of peritoneum	XI	0·02	2·6 (1·2-5·1)	0·163
G1/G2	M15	Polyarthrosis	XIII	0·02	2·8 (1·2-5·7)	0·163
G1/G2	N13	Obstructive and reflux neuropathy	XIV	0·004	3·6 (1·6-7·7)	0·122
G1/G2	N17	Acute renal failure	XIV	0·03	1·7 (1·1-2·5)	0·180
G1/G2	N28	Other disorders of the kidney and ureter, not elsewhere classified	XIV	0·02	2·2 (1·1-4·1)	0·177
G1/G2	N40	Hyperplasia of prostate	XIV	0·01	2·0 (1·2-3·5)	0·163
G1/G2	O26	Maternal care for other conditions predominantly related to pregnancy	XV	0·005	3·5 (1·5-7·4)	0·122
G1/G2	O36	Maternal care for other known or suspected foetal problems	XV	0·01	3·3 (1·4-7·4)	0·142
G1/G2	U07	Emergency use of U07	XXII	0·002	2·5 (1·4-4·2)	0·122

Supplementary Table 3: Level 2 International Classification of Disease, Version 9 and 10 (ICD-9 and ICD-10) codes for which a potential association with *APOL1* risk alleles was indicated by the phenome-wide screen using data from UK Biobank participants with African ancestry.

Chapter	Level 2 codes analysed	Level 2 codes with P<0·05 and FDR<20% (%)	p
**I/U07**	**11**	**5 (45·5%)**	**0·002**
II	13	0 (0%)	0·18
III	7	0 (0%)	0·33
IV	14	2 (14·3%)	0·67
V	8	1 (12·5%)	0·97
VI	10	0 (0%)	0·25
VII	15	0 (0%)	0·16
VIII	1	0 (0%)	0·71
IX	27	4 (14·8%)	0·67
X	11	2 (18·2%)	0·54
XI	31	5 (16·1%)	0·52
XII	8	0 (0%)	0·30
XIII	25	1 (4·0%)	0·23
XIV	26	4 (15·4%)	0·62
XV	10	2 (20·0%)	0·45
XVI	0	0 (0%)	N/A
XVII	0	0 (0%)	N/A
Total	217	26 (12·0%)	

Supplementary Table 4: Counts of Level 2 ICD codes for which a potential association with the G1/G2 genotype was indicated by the phenome-wide screen, by coding chapter. P values are for an excess of phenotypes with an association in each chapter calculated using z-score tests.

Genotype	n (total)	ICD codes per participant	p
G0/G0	2,853	6·5	
G0/G1	2,273	7·1	0·85
G0/G2	1,219	6·8	0·79
G1/G1	644	6·7	0·59
**G1/G2**	320	**8·7**	**0·0003**
G2/G2	153	6·3	0·18

Supplementary Table 5: International Classification of Disease, Version 9 and 10 (ICD-9 and ICD-10) codes per participant, comparing *APOL1* genotypes containing risk variants relative to G0/G0. Adjusted for age, sex, body mass index, Townsend deprivation index, and principal components 1-4. P values ≤0·05 are shown in bold.

		Primary analysis model			Model 2		
**ICD code**	**ICD code descriptor**	**p-value**	**Odds ratio (95% CI)**	**False discovery rate**	**p-value**	**Odds ratio (95% CI)**	**False discovery rate**
A09	Diarrhoea and gastro-enteritis of presumed infectious origin	**0·01**	**1·9 (1·1-3·1)**	**0·16**	**0·001**	**2·9 (1·5-5·5)**	**0·10**

B96	Other bacterial agents as the cause of diseases classified to other chapters	**0·02**	**1·8 (1·1-2·8)**	**0·16**	**0·001**	**2·8 (1·5-5·1)**	**0·08**
E87	Other disorders of fluid, electrolyte, and acid-base balance	0·06	1·5 (1·0-2·4)	0·35	**0·001**	**2·7 (1·5-4·7)**	**0·08**
J96	Respiratory failure, not elsewhere classified	**0·02**	**2·1 (1·1-3·8)**	**0·17**	**0·005**	**3·2 (1·4-7·1)**	**0·18**
N17	Acute renal failure	**0·02**	**1·6 (1·1-2·5)**	**0·18**	**0·005**	**2·2 (1·3-3·7)**	**0·18**
N32	Other disorders of the bladder	0·03	1·8 (1·0-3·0)	0·22	**0·005**	**2·6 (1·3-5·0)**	**0·18**

Supplementary Table 6: Level 2 International Classification of Disease, Version 9 and 10 (ICD-9 and ICD-10) for which a potential association with the G1/G2 interaction was indicated by the phenome-wide screen using Model 2. P-values, odds ratios, and false discovery rates for each code using the Primary Analysis models displayed for comparison.

**Number of variants**	**n (total)**	**uACR >3 mg/mmol or eGFR <60 mL/min/1·73m^2**	**uACR >3 mg/mmol**	**eGFR <60 mL/min/1·73m^2**
0 variants	2,853	276 (9·7%)	205 (7·2%)	91 (3·2%)
1 variant	3,492	373 (10·7%)	284 (8·1%)	120 (3·4%)
2 variants	1,117	159 (14·2%)	122 (10·9%)	58 (5·2%)

Supplementary Table 7: indicators of CKD among UK Biobank participants with African ancestry, comparing rates by number of *APOL1* variants.

**Genotype**	**n (total)**	**uACR >3 mg/mmol or eGFR <60 mL/min/1·73m^2**	**uACR >3 mg/mmol**	**eGFR <60 mL/min/1·73m^2**
G0/G0	2,853	276 (9·7%)	205 (7·2%)	91 (3·2%)
G0/G1	2,273	239 (10·5%)	184 (8·1%)	76 (3·3%)
G0/G2	1,219	134 (11·0%)	100 (8·2%)	44 (3·6%)
G1/G1	644	93 (14·4%)	74 (11·5%)	29 (4·5%)
G1/G2	320	48 (15·0%)	37 (11·6%)	17 (5·3%)
G2/G2	153	18 (11·7%)	11 (7·2%)	12 (7·8%)

Supplementary Table 8: indicators of CKD among UK Biobank participants with African ancestry, comparing rates by *APOL1* genotype.

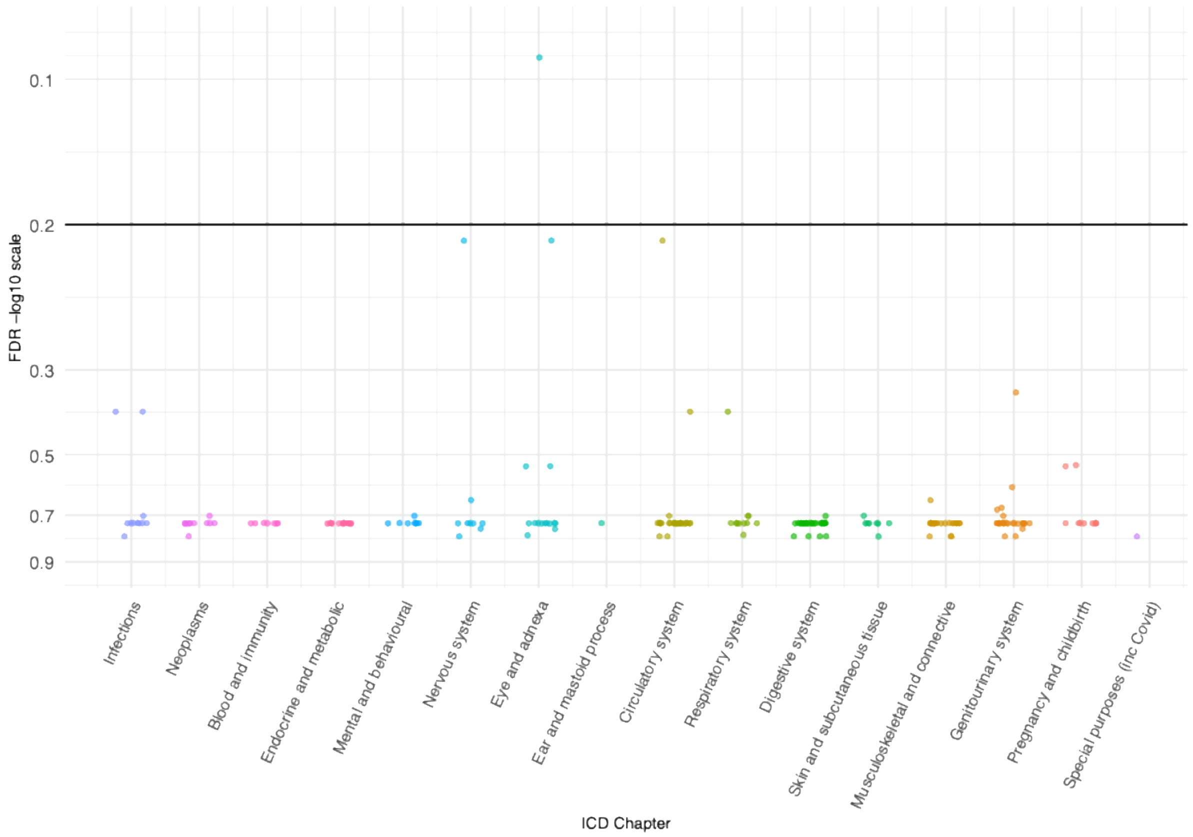

Supplementary Figure 1: Plot of false discovery rate values showing associations between each ICD Level 2 code tested in the phenome-wide data and the *APOL1* G0/G2 genotype.

Identification of end stage kidney disease

End stage kidney disease (ESKD) as of September 2022 was defined as reaching CKD stage G5 or the requirement for kidney replacement therapy, using ICD-10 codes for hospital admission, or Office of Population Censuses and Surveys Classification of Surgical Operations and Procedures, Version 4 (OPCS4) codes for operative procedures. Participants were considered to have developed ESKD if ICD-10 codes E853, N165, N180, N185, Q601, T824, T861, Y602, Y612, Y622, Y841, Z490, Z491, Z492, Z940, Z992 , or OPCS4 codes L741, L742, L743, L744, L745, L746, L748, L749, M012, M013, M014, M015, M018, M019, M023, M084, M172, M174, M178, M179, X401, X402, X403, X404, X405, X406, X407, X408, X409, X411, X412, X418, X419, X421, X428, X429, X431 had been recorded, or if ICD-10 codes N180 or N185 appear in any position in their death record.

Sensitivity analysis

The ability to detect associations between the different haplotype combinations and phenotype codes using the Biobank data set for participants with African Heritage with the logistic regression model described above was estimated by simulation. Sensitivity was estimated by assigning phenotypes at random and finding the minimum odds ratio > 1 with nominal $p < 0{\cdot}05$ observed for each haplotype combination. The same model was used as for the main analysis. Firth's bias-reduced logistic regression was used to test the association of each phenotype with the six *APOL1* haplotype combinations. Covariates were age, sex, Townsend deprivation index, hypertension, diabetes and the first 10 UK Biobank principal

components. Given the fixed sample size, the main factors determining power in this analysis are the numbers of participants with each phenotype code and the frequency of the haplotype combinations. The deciles of the counts of phenotype codes were obtained and for each decile 1000 replicate analyses were conducted with phenotypes assigned at random to participants to obtain a range of odds ratios and p values. For each decile and haplotype combination the minimum observed odds ratio with nominal $p < 0{\cdot}05$ was taken as an estimate of the sensitivity of the model to detect an association with that haplotype combination and that number of affected participants.

The counts of participants with each phenotype were obtained and for each decile of the counts distribution an estimate was made of the minimum odds ratio > 1 with nominal $p < 0{\cdot}05$ that could be obtained with the model and the number of participants with each haplotype combination (Supplementary Figure 2). As expected, the minimum detectable odds ratio was inversely related to the number of participants with each haplotype combination. The G1/G2 combination which had the most associations with phenotype after the FDR correction had the second-highest minimum detectable odds ratio, indicating that the excess of associations with this haplotype combination was not due to a relatively high power to detect associations with participants with this combination. Conversely G2/G2 had a much lower power than other haplotype combinations and it is possible that associations with this haplotype combination have been underestimated due to lack of power.

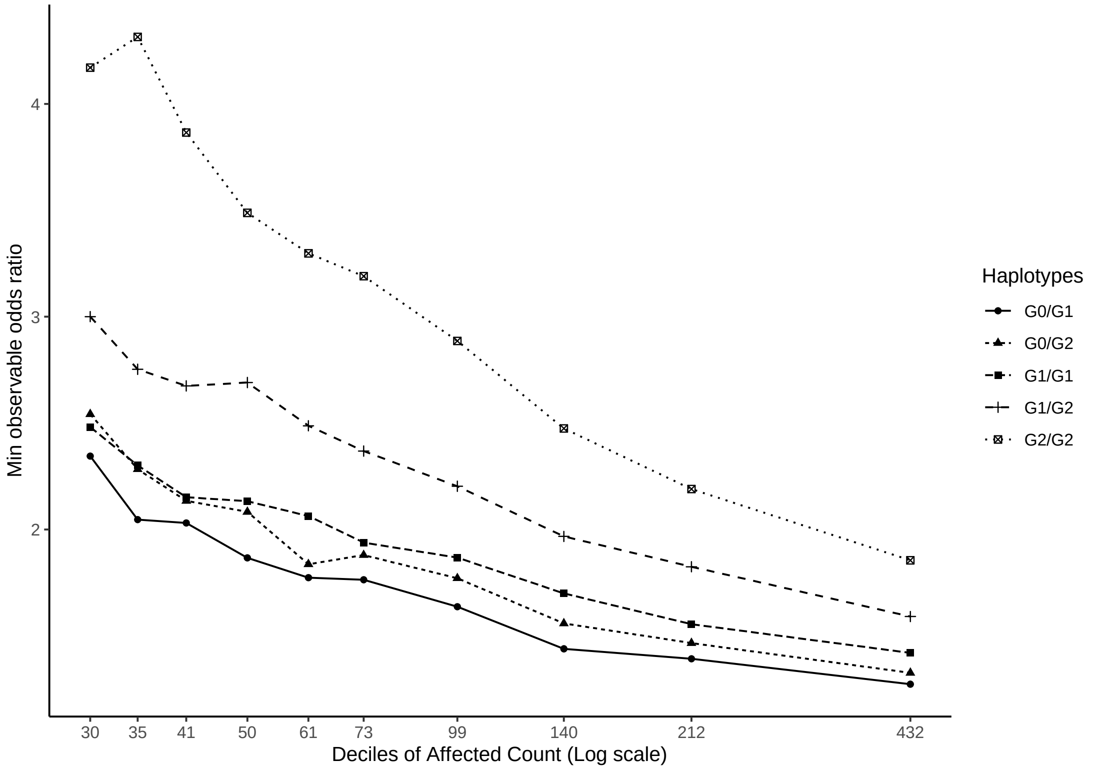

Supplementary Figure 2. Minimum odds ratios for each haplotype combination and decile of affected counts with p < 0·05. Odds ratios and p values were generated by applying the phenome wide regression model to a dummy phenotype with 'Count Affected' numbers of participants being randomly assigned as cases. The affected count represents the deciles of the distribution of numbers affected.

Interaction analysis and epistasis

Phenome-wide scan data (Table 2) was obtained considering the six observed *APOL1* genotypes as a single independent variable with six levels. For each ICD code, the effect of the five non-G0/G0 genotypes was tested against G0/G0. In addition, we formally tested for interaction in the G1/G2 genotype by running a model (Model 2) with genotype at the G1 locus, genotype at the G2 locus, and the G1/G2 interaction term as separate independent variables. These three variables produce the same total of five non-reference levels as the primary analysis model, however the null hypotheses being tested differs: in Model 2, the effect of the G1/G2 interaction was estimated relative to the effect expected by combining the independent effects of heterozygosity at the G1 locus and heterozygosity at the G2 locus, rather than relative to G0/G0. All other covariates were the same in both models. Model 2 identified six ICD codes showing an association with the G1/G2 interaction, compared with 26 that were associated with the G1/G2 genotype in the primary analysis model (Supplementary Table 8). Four of these six ICD codes were identified by both models.

The possible reasons why an interaction effect was not detected for 22 of the 26 that showed a significant main effect of G1/G2 are (1) that there is no interaction effect; (2) that there is less power to detect an interaction effect than to detect the main effect of G1/G2. We argue that lack of power is likely to have significantly reduced our ability to detect interactions. The power of Model 2 to detect an association is expected to be lower for two reasons.

First, in our primary analysis model, the effect of the G1/G2 genotype is a measure of the independent effects of heterozygosity and the G1 locus, heterozygosity and the G2 locus, and the interaction between G1 and G2 in participants with the G1/G2 genotype. It is the sum of these effects that are significantly associated with ICD codes. Model 2 detects associations with each of these factors independently. The 26 associations with the G1/G2 genotype identified in the primary analysis model all have odds ratios >1. In this model, the mean odds ratios for the G0/G1 genotype (i.e. heterozygosity at the G1 locus alone) and the G0/G2 genotype (heterozygosity at the G2 locus alone) are 1·14 and 1·08 respectively, indicating that the main effects of the genotypes at each locus are contributing positively to the overall effect, whereas the mean odds ratios for heterozygosity at the G1 locus and heterozygosity at the G2 locus for the six associations detected in Model 2 are 0.85 and 0.78. These differences in odds ratios between models is significant: $p = 0.0006$ and $p = 0.0001$ for heterozygosity at the G1 and G2 locus respectively (paired t-test). This indicates that in our primary analysis model, the independent effects of G1 and G2 are in the same direction as the interaction effect, enhancing the power to detect associations with the G1/G2 genotype. Conversely, the mean odds ratios for heterozygosity at the G1 and G2 loci in Model 2 are <1, even though the G1/G2 interaction has an odds ratio of >1, suggesting that there is power to detect a positive interaction effect when the individual effects are negative.

Second, comparing the G1/G2 interaction in Model 2 with a model-predicted combined effect rather than the risk estimated from a single common genotype adds uncertainty and therefore reduces power. This additional uncertainty is reflected in the mean standard error of the G1/G2 interaction in Model 2 (0·43) was larger than that for the G1/G2 genotype in the primary analysis model (0·33) for the 26 ICD codes associated with the G1/G2 genotype

(paired t-test, $p = 9x10^{-15}$, showing that Model 2 would require more samples than the primary analysis model to detect the same effect.

In the primary analysis model, no associations with ICD codes were detected for the G1/G1 genotype (compared to 26 for the G1/G2 genotype) despite their being 644 G1/G2 participants and only 320 G1/G2 participants. This is suggestive of an epistatic interaction. The data from Model 2 indicates that some associations with the G1/G2 interaction occur, and the lower power to formally detect associations in this model suggests that more would be detected with larger numbers of samples.
